# Fertilization drives distinct biotic and abiotic factors in regulating functional groups of protists in a 5-year fertilization system

**DOI:** 10.3389/fmicb.2022.1036362

**Published:** 2022-12-05

**Authors:** Siyu Zhang, Haifang Zhang, Hongmei Liu, Hui Wang, Weiming Xiu, Gang Li, Guilong Zhang, Zhongkai Zhou, Na Jiang, Hao Zhang, Jianning Zhao, Dianlin Yang

**Affiliations:** Agro-Environmental Protection Institute, Chinese Academy of Agricultural Sciences, Tianjin, China

**Keywords:** Protist, fertilization, community composition, functional groups, edaphic factors, trophic interaction

## Abstract

**Introduction:**

Protists play an important role in nutrient cycling, microbiome stability and soil fertility maintenance. However, the driving force of protistan functional groups remains poorly understood in agricultural ecosystems.

**Methods:**

We investigated the impacts of fertilization regimes on the diversity, composition and functional groups of protists and further disentangled the effects of multiple factors shaping the community composition of functional groups in a 5-year fertilization regime (CK, no fertilization; M, organic fertilization; MNPK, combined inorganic and organic fertilization; NPK, inorganic fertilization).

**Results:**

Fertilization significantly changed the community composition of protists rather than diversity. The MNPK treatment significantly increased the relative abundance of phototrophs and decreased that of the parasites and consumers. Partial least squares path modeling indicated that fertilization indirectly regulated protistan consumers via changes in the P content, which affected the composition of consumers mainly by regulating fungal community composition. Soil moisture (SM) and available phosphorus (AP) were identified as the top predictors for the composition of parasites, and the composition of phototrophs was mainly affected by SM, indicating that parasites and phototrophs were more sensitive to abiotic factors in the fertilization system.

**Discussion:**

Taken together, our findings highlight that fertilization significantly affects the composition of functional groups of protists and their biotic or abiotic regulatory processes, which have implications for the potential changes in their ecosystem functions for soil management systems.

## Highlights

- The functional groups of protists and specific taxa sensitive to fertilization were identified.
- The composition of protistan consumers were directly and mainly affected by the fungal community composition.
- Fertilization indirectly controlled protistan parasites *via* changes in available phosphorus (AP).
- Phototrophs were mainly affected by soil moisture (SM).

## Introduction

Protists, as microscopic eukaryotes, are an important but generally ignored component of soil food webs. Protists are of major importance for ecosystem stability and providers of ecosystem services, such as nutrient cycling, population control and carbon sequestration (Geisen, 2016; Lee et al., 2022). Furthermore, protists are useful in applied research as bioindicators of soil quality (Geisen et al., 2018). Fertilization is a common and effective practice used to increase crop yields, but the intensive application of fertilizers has some negative effects on soil microbial function (Xie et al., 2022). Protist communities are more sensitive to fertilization than other microorganisms (Zhao et al., 2019, 2020). Therefore, understanding the taxonomic and functional traits of protists responding to fertilization is critical to the sustainability of an agroecosystem.

Organic fertilizer application is considered an ideal agricultural practice that produces good crop yields with minimal impacts on ecological factors (Mader et al., 2002; Zhang et al., 2022). Recent studies have reported that fertilization regimes affect the taxonomic composition and function of protists (Zhao et al., 2020; Sun et al., 2021), while the fundamental mechanisms and driving forces regulating protist community composition and function in agricultural ecosystems remain unclear. Fertilization changes the physicochemical condition of the soil, which is a key factor influencing the protist community (Oliverio et al., 2020). Furthermore, the effects of the environment on coexistence within biological communities have been explored (Li et al., 2017). However, we have limited knowledge of the impacts of fertilization practices on protists through trophic interactions. Such knowledge is necessary for the development of agricultural fertilization management to improve the microbial role in regulating ecosystem function.

Soil protist communities form a dynamic hub in the soil microbiome (Xiong et al., 2018), suggesting that microbial taxa strongly co-occur with each other (Toju et al., 2018). This result also indicated that trophic food web interactions could be a key driving force in shaping the protist community in addition to abiotic factors. Bacteria and fungi regulate protistan functional groups due to the bottom-up effects through trophic regulations (Nguyen et al., 2021), but the microbial processes under fertilization systems remain unclear. Suleiman et al. (2019) indicated that organic fertilization increased the connections among categories of primary decomposers (bacteria and fungi) and predators (protozoa and microfauna) and differences in potential function, while the fertilization-regulated protist community through the changed edaphic factor and other trophic interactions should be more thoroughly investigated. Further explicit research on protists is essential to the development of agricultural fertilization management of soil processes.

Our study estimated the taxonomic and functional compositions of protists and then evaluated how fertilization changed the soil environment by influencing biotic factors (bacterial and fungal community composition) in shaping protistan functional groups. We selected a 5-year fertilization experiment in the north plain of China. The protist community was quantified and identified into 3 functional groups based on 18S rRNA gene amplicon sequencing to characterize the composition of the protistan community and their functional groups. The edaphic and biotic (bacterial and fungal community composition) factors were used to assess the effects of abiotic and biotic factors in shaping protistan functional groups. We hypothesized that (1) fertilization shifts the protistan community composition and functional groups and (2) functional groups had different responses to biotic and abiotic factors under different fertilization treatments according to their specific functional traits in ecosystems.

## Materials and methods

### Experimental design and sampling

The fertilizer experimental site used in this study was established on a wheat (*Triticum aestivum* cv. Jimai22)-maize (*Zea mays* cv. Jiyuan 169) rotation field (39°21′N, 117°12′E) in Tianjin, China, in 2016. The site has a typical temperate continental monsoon climate with a mean annual temperature of 11.6°C and a mean annual precipitation of 606 mm. The soil is sandy loam (FAO Soil Classification), classified as alkaline fluvo-aquic soil in China. Four fertilization treatments were compared in a completely randomized block design with six replicates (each plot was 96 m^2^): (1) CK, unfertilized control; (2) NPK, inorganic fertilization; (3) M, organic fertilization; and (4) MNPK, combined organic–inorganic fertilization. All treatments received 200 kg N ha y^−1^, 100 kg P_2_O_5_ ha y^−1^ and 100 kg K_2_O ha y^−1^ for wheat and maize based on the recommended fertilization rate for the Chinese annual double cropping system. The inorganic nitrogen, phosphorus and potassium were urea, superphosphate and potassium sulfate, respectively. The organic fertilizer contained 2.0% nitrogen, 1.5% phosphorus and 1.5% potassium. Organic fertilization was applied all at once before sowing. In the MNPK treatment, 30% of mineral N fertilizer was substituted by organic N. Approximately 10 t ha^−1^ of organic fertilizers were applied manually for the M treatment. For the other two fertilization treatments, 60% N, 100% P, and 100% K were basally applied before sowing, and 40% N was applied as dressing fertilizer at the joint stage. The aboveground crop biomass was removed after harvest. The soil properties in each treatment plot are provided in Supplementary Table S1.

Bulk soil samples under different fertilization treatments were collected in September 2020. Twenty soil cores were collected from the 0–20 cm depth and mixed thoroughly at each plot and then sieved with 2-mm mesh to remove roots and other litters. The soil was divided into two parts and stored at either 4°C for soil property measurements or − 80°C for DNA extraction.

### Molecular analysis

Soil DNA was extracted from 0.25 g soil using FastDNA Spin Kits (MP Biomedical, Santa Ana, California, United States). The 18S rRNA gene of protists was determined using the primer TAReuk454FWD1F (5’-CCAGCASCYGCGGTAATTCC-3′) and the reverse primer TAReukREV3R (5’-ACTTTCGTTCTTGATAGA-3′). Briefly, the PCR protocol for protists was conducted with the following procedure: 94°C for 3 min, followed by 27 cycles at 95°C for 30 s, 55°C for 30 s and 72°C for 45 s and a final extension at 72°C for 10 min. The primers 338F (5’-ACTCCTACGGGAGGCAGCAG-3′) and 806R (5’-GGACTACHVGGGTWTCTAAT-3′) were used to amplify the bacteria-specific V3-V4 region of the 16S rRNA gene for bacterial analysis. Briefly, the PCR protocol for bacteria was conducted with the following procedure: 94°C for 5 min, followed by 28 cycles at 94°C for 30 s, 55°C for 30 s and 72°C for 60 s and a final extension at 72°C for 7 min. The fungal-specific ITS1 region was amplified with the primer pair ITS1F (5’-CTTGGTCATTTAGAGGAAGTAA-3′) and ITS2R (5’-GCTGCGTTCTTCATCGATGC-3′). PCR for fungal sequences was conducted for 3 min at 95°C, followed by 35 cycles of 30 s at 95°C, 30 s at 59.3°C and 45 s at 72°C, and a final extension at 72°C for 10 min. Sequencing data including bacteria, fungi and protists have been submitted to the NCBI Sequence Read Archive (accession No. PRJNA898295, 898,261 and 893,190).

VSEARCH tools were used to detect and remove chimeras (Rognes et al., 2016). Sequences were assigned to operational taxonomic units (OTUs) based on a 97% level of similarity using UPARSE 7.1 (Edgar et al., 2011). OTUs lacking more than two sequences were removed. For 18S data, taxonomic assignment was performed using the Protist Ribosomal Reference (PR2) database (version 4.5). The OTU tables were resampled to a minimum number of sequences from each sample of 13,797 for protists. The protistan OTU tables defined as Fungi, Metazoa, Rhodophyta and Streptophyta were removed. Four main functional groups were manually assigned to the protist community: consumers, phototrophs, parasites and others. Protist lineages in the supergroup Rhizaria and other supergroups were classified according to the classifications of Dumack et al. (2020) and Nguyen et al. (2020), respectively.

Nine edaphic factors were measured using standard testing methods, including soil pH, soil moisture (SM), soil organic matter (SOM), total nitrogen (TN), available phosphorus (AP), total phosphorus (TP), dissolved organic carbon (DOC), ammonium-nitrogen (NH_4_^+^-N) and nitrate nitrogen (NO_3_^−^-N), as reported in a previous study (under review).

### Statistical analyses

Analysis of differences in the relative abundance of supergroups and functional groups under the different fertilization treatments was based on ANOVA (Duncan). Principal coordinate analysis (PCoA) was used to evaluate the difference in protistan community composition across fertilization treatments using the ‘vegan’ package based on the Bray–Curtis dissimilarity. PERMANOVA was used to determine the similarity between the composition of protistan communities among treatments. The Mantel test was used to evaluate the correlations of biotic and abiotic factors with the soil protist functional community. The biotic factors (bacterial and fungal community composition) were represented by the first axis of PCoA. Spearman correlation was conducted to evaluate the relationship of the protist community with individual bacterial and fungal taxa at the class level. To explore the direct and indirect relationships among fertilization, edaphic, biotic factors and the composition of protistan functional groups (based on the first axis of PCoA), directed graphs of the partial least squares path model (PLS-PM) analysis were conducted with the package ‘plspm’ (Wen et al., 2022). Notably, this is an exploratory data analysis technique that may be applied to any kind of dataset and has little limitation regarding data independence and normality. The variables with loadings <0.7 were removed, and then the final models were built with the remaining variables. After adjustment, the goodness-of-fit values of the models for consumers, parasites and phototrophs were 0.65, 0.73 and 0.54, respectively, indicating a high degree of confidence. R software (4.5.3) was used for statistical analysis.

## Results

### Diversity and composition of protistan communities under different fertilization treatments

**Figure 1:**
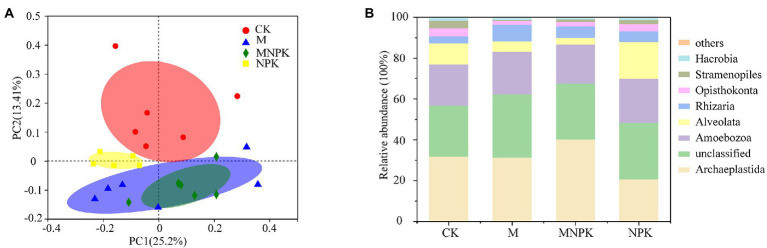
Principal coordinate analysis (PCoA) grouped by fertilization treatments based on the Bray–Curtis distance **(A)**. Composition of protists across different fertilization treatments at the supergroup level **(B)**.

Fertilization had no significant impacts on the alpha diversity (Shannon, PD and richness index) of protists (Supplementary Table S2). Principal coordinate analysis (PCoA) with Bray–Curtis distance matrixes revealed that protist community structure was significantly affected by fertilization (ADONIS, *p* < 0.001; Figure 1A). PERMANOVA indicated that the protistan community differed significantly between every two treatments except for the M and MNPK treatments (*p* > 0.05; Supplementary Table S3). Archaeplastida and Amoebozoa were the dominant supergroups of protists (Figure 1B). The NPK treatment increased the relative abundance of Alveolata, while it decreased the relative abundance of Archaeplastida compared to the MNPK treatment (Supplementary Figure S1).

### Functional groups of protists under different fertilization treatments

**Figure 2:**
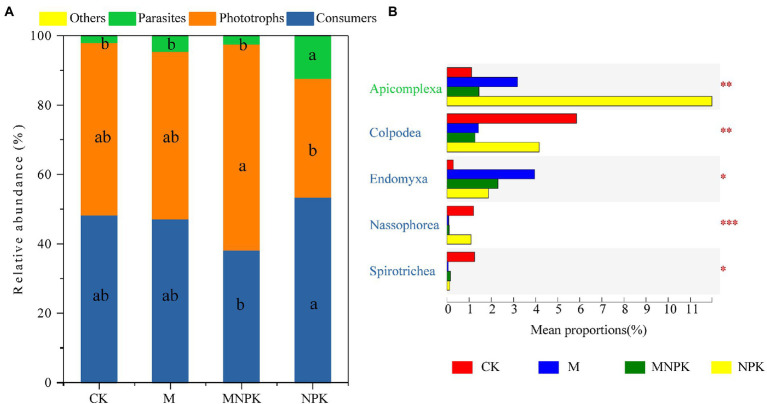
**(A)** Changes in the relative abundance of functional groups. Letters indicate significant differences in the relative abundance among functional groups under the effects of fertilization (one-way ANOVA, Duncan’s test; *p* < 0.05). **(B)** Boxplots of the percent relative abundance of soil protistan groups at the phylum level; colored labels represent corresponding functional categories. Difference analysis of soil protistan groups at the phylum level was identified based on Kruskal–Wallis analysis with a *post hoc* test (significance is indicated by *p < 0.05, **p < 0.01, ***p < 0.001).

Consumers, phototrophs and parasites were the dominant functional groups of protists under the different fertilization treatments (Figure 2A). The MNPK treatment significantly increased the relative abundance of phototrophs compared to the NPK treatment, while those of consumers and parasites followed the opposite trends (Figure 2A). Further analysis of protistan functional groups at the class level showed that the fertilization treatments had significant effects on Colpodea, Endomyxa, Nassophorea and Spirotrichea of consumers, as well as on Apicomplexa of parasites (Figure 2B).

### Biotic and abiotic factors drive protistan communities under different fertilization treatments

**Figure 3:**
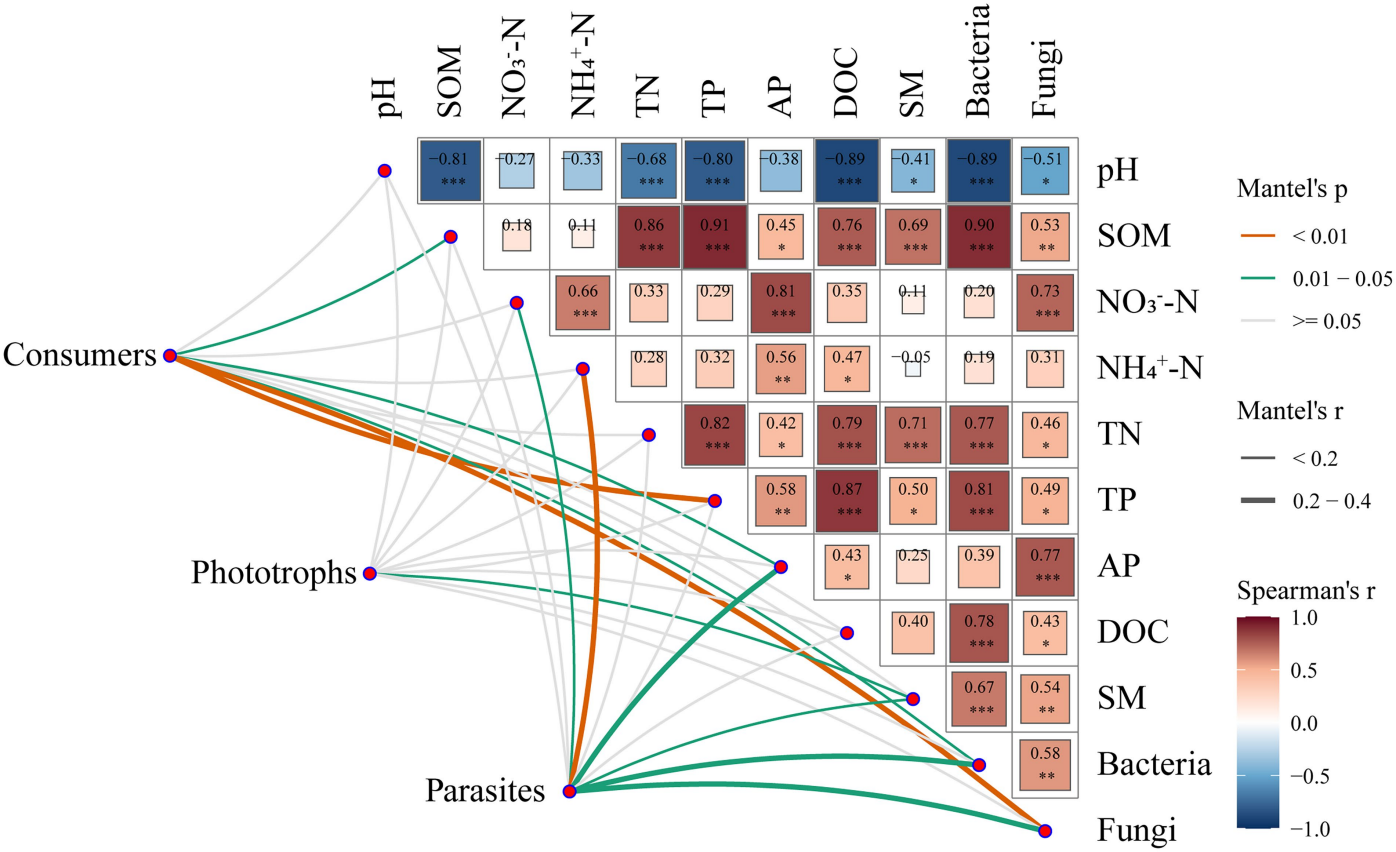
Pairwise comparisons of biotic and abiotic factors are shown, with a color gradient denoting Spearman’s correlation coefficients. Protistan functional groups were related to each biotic and abiotic factor by Mantel tests. The edge width corresponds to Mantel’s *r* statistic for the corresponding distance correlations, and the edge color denotes the statistical significance based on 9,999 permutations.

We also identified the important factors constructing the composition of the functional groups of protists (Figure 3). Mantel tests showed that the fungal community composition was the most effective factor for the community composition of consumers (*r* = 0.38, *p* < 0.01), followed by TP (*r* = 0.32, *p* < 0.01), AP (*r* = 0.20, *p* < 0.05), SOM (*r* = 0.20, *p* < 0.05) and the bacterial community composition (*r* = 0.19, *p* < 0.05; Figure 3). The edaphic factor SM (*r* = 0.17, *p* < 0.05) was the best predictor of the community composition of phototrophs. For the composition of parasites, NH_4_^+^-N was the best predictor (*r* = 0.38, *p* < 0.01), followed by the fungal community composition (*r* = 0.27, *p* < 0.05), AP (*r* = 0.24, *p* < 0.05), bacterial community composition (*r* = 0.22 *p* < 0.05), SM (*r* = 0.19, *p* < 0.05) and NO_3_^−^-N (*r* = 0.15, *p* < 0.05).

**Figure 4:**
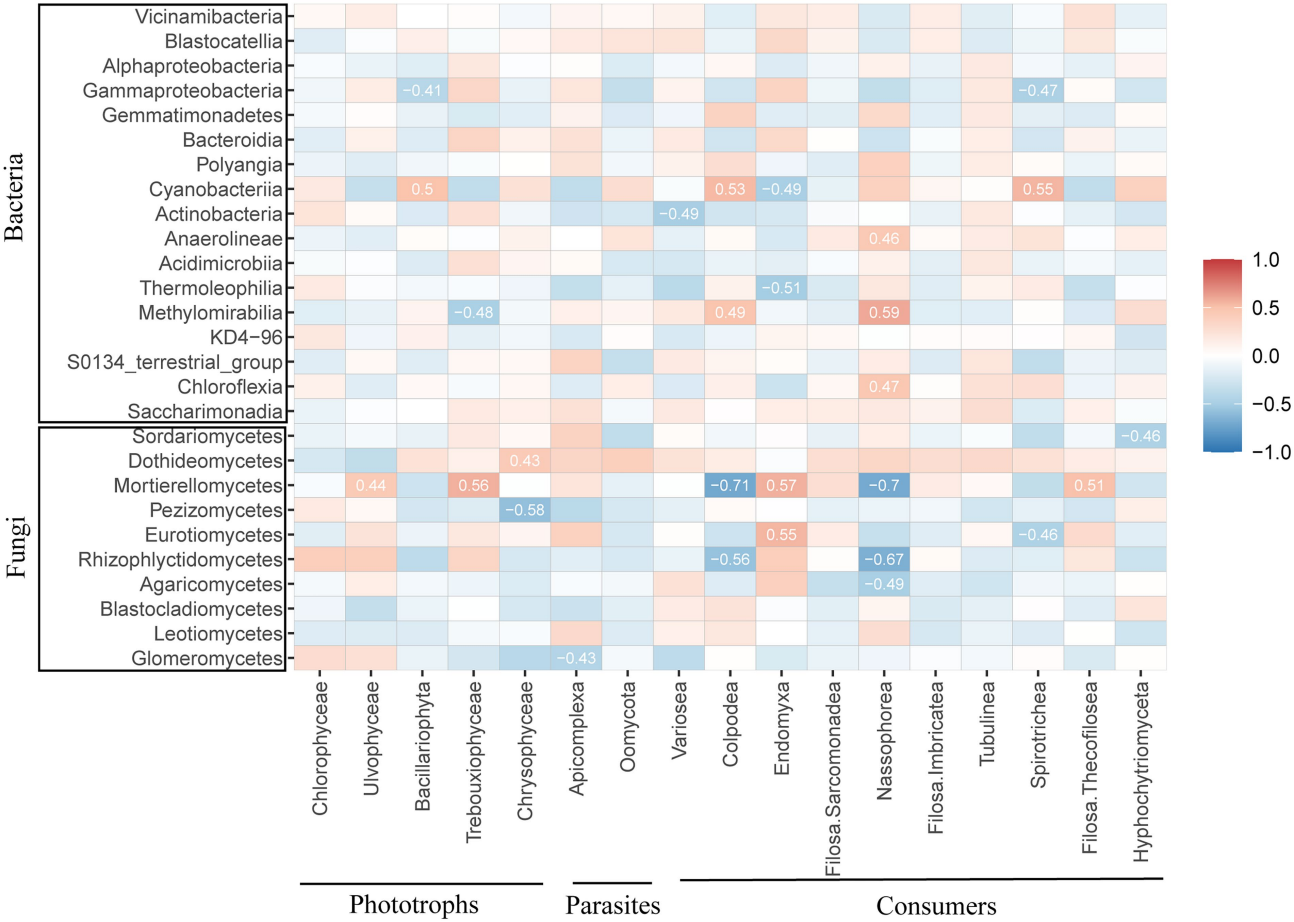
Correlation analysis of the relative abundance of protists with bacteria and fungi at the class level. Blue indicates a negative correlation, red represents a positive correlation, and the strength of the color reflects the strength of the correlation.

Spearman correlations between the protistan community and the biotic factors of the bacterial and fungal communities at the class level were analyzed to confirm the specific biotic factor (Figure 4). Specifically, the bacterial taxa Gammaproteobacteria, Cyanobacteria, and Methylomirabilia and the fungal taxa Dothideomycetes, Mortierellomycetes and Pezizomycetes were correlated with phototrophs. Glomeromycetes were significantly and negatively correlated with Apicomplexa of parasites. The variance taxa of the consumers Colpodae, Endomyxa, Nassophorea and Spirotrichea under different fertilization treatments were correlated with Gammaproteobacteria, Cyanobacteria and Methylomirabilia of the bacterial taxa. The fungal taxa Mortierellomycetes, Eurotiomycetes and Rhizophlyctidomycetes were related to the consumer community.

**Figure 5:**
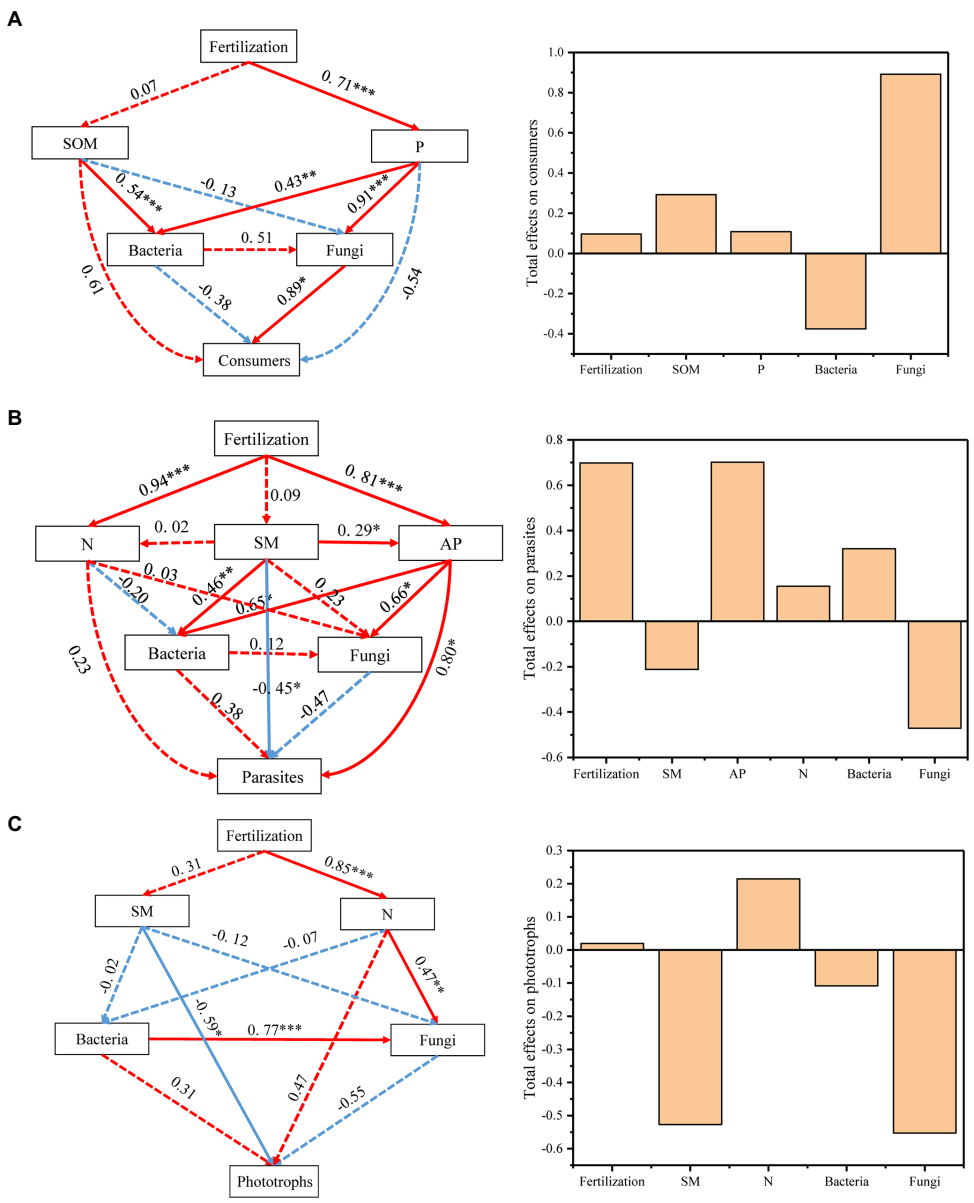
Effects of various factors on the composition of consumers **(A)**, parasites **(B)** and phototrophs **(C)**. Numbers adjacent to arrows are indicative of the “direct effects” value. Continuous and dashed lines indicate significant and nonsignificant relationships, respectively. Red and blue lines indicate positive and negative relationships, respectively. The total effects indicate direct plus indirect effects calculated by partial least squares path modeling (PLS-PM). Significance is indicated by *p < 0.05, **p < 0.01, ***p < 0.001.

To better integrate the complex interrelationships among fertilization, edaphic factors, biotic factors (bacterial and fungal composition) and individual protistan functional groups, we constructed a partial least squares path model (PLS-PM; Figure 5). The indirect effects of the fertilization treatments on the composition of consumers were caused by changes in the P nutrient and fungal community composition (Figure 5A). Fungal community composition was the most important factor for the soil protistan consumer community, and its total effect was 0.89. SM and AP significantly and directly affected the composition of parasites, and fertilization and SM indirectly affected the parasites by directly regulating the AP content (Figure 5B). Fertilization and AP were the most important factors for parasites, and their total effects were both 0.70 (Figure 5B). For phototrophs, SM was the most effective factor. The fungal community composition and SM contributed the most to phototrophs, with total effects of −0.55 and − 0.53, respectively (Figure 5C).

## Discussion

### Protistan community and functional group responses to fertilization treatments

Protists play an important role in plant nutrient availability and microbiome stability (Geisen et al., 2018), though they have been generally ignored in previous studies that focused more on bacteria and fungi. Recently, studies have provided evidence that fertilization, which is important for agricultural management to increase productivity, could remarkably influence the protistan community and its ecosystem function (Xiong et al., 2018; Sun et al., 2021). Our research indicated that fertilization changed the composition of protists rather than their diversity (Figure 1; Supplementary Table S2). Li et al. (2021) also found the same results that inorganic fertilization had no significant effects on diversity but did have significant effects on specific taxa. The stable diversity in all treatments is partially due to the relatively high proportion of taxa belonging to the core microbiome (Lentendu et al., 2014). Archaeplastida, Amoebozoa, Alveolata and Rhizaria were core supergroups in our study, which accounted for approximately 65.70–68.43% of every treatment (Figure 1B). Furthermore, the results emphasized that community analyses have to go beyond diversity to better understand underlying ecological patterns (Shade, 2017). In our study, Archaeplastida, Alveolata and Hacrobia were more sensitive to fertilization (Supplementary Figure S1), indicating their adaptation to specific conditions. For example, the relative abundance of Archaeplastida was decreased under the NPK treatment compared to the MNPK treatment (Supplementary Figure S1). This result could be illustrated by the increased SM after organic fertilizer application (both the M and the MNPK treatments), which was the key factor influencing phototrophs (Figure 3). Moreover, Nguyen et al. (2021) found that mean annual precipitation (MAP) and TN were the best drivers of the composition of phototrophs in natural soil ecosystems, which further confirmed our results.

### Fertilization drives the edaphic and biotic factors in regulating protistan functional groups

As consumers in soil food webs, protists play a major role in maintaining fertility through predatory action (Bass and Cavalier-Smith, 2004). Conversely, bacteria and fungi (as the main food source of protists), combined with biotic characteristics of the environment, shape the protist composition and functional groups jointly (Geisen et al., 2017). Organic fertilizer amendments often enhance the relative abundance of predators, which can be explained by the fact that organic fertilizers provide a wider resource spectrum than chemical fertilizers (Xiong et al., 2018). The MNPK treatment significantly decreased the relative abundance of consumer groups compared to the NPK treatment in our study (*p* < 0.05; Figure 2A). Our PLS-PM results revealed that the fungal community composition had the direct and highest total effects on the protistan consumer groups (Figure 5A), which further identified trophic interactions as a key contributor to the distribution pattern of protistan consumers (Nguyen et al., 2021). At the class level, *Colpodea* and *Nassophorea* were consumers reduced by organic fertilization, while a diverse pattern was observed on *Endomyxa* (Figure 2B). The results suggested that specific taxa have a low tolerance to fertilization disturbance (Sun et al., 2021). Furthermore, this may be explained by feeding differences between protist predators and their prey (Karakoc et al., 2020). For example, *Endomyxa* had a positive correlation with *Mortierellomycetes* and *Eurotiomycetes*, while *Colpodea* and *Nassophorea* had a negative correlation with *Mortierellomycetes* and *Eurotiomycetes*. Together, our results highlight that the fungal community composition is the main factor that regulates consumers in different fertilization management systems.

Parasitic groups of protists can regulate animal communities and release nutrients into the soil (Mahe et al., 2017). The relative abundance of parasite groups of protists was significantly increased in the NPK treatment compared to the other treatments (Figure 2A), which was dominated by the parasitic Apicomplexa (Figure 2B). Our results showed that the AP content and fertilizer type were the most dominant factors regulating the parasitic groups (Figure 5). Lambers et al. (2018) found that roots of mycorrhizal plants may not be as effective at acquiring P when P availability is very low, but they are better defended against Oomycetes. This indicated that AP directly influenced the composition of parasites. The changes in protistan parasites by fertilization may also be explained by the changes in the main hosts of parasitic protists after long-term inorganic fertilization, which can then indirectly affect the patterns of parasites (Schulz et al., 2019). Apicomplexa are common parasites of soil invertebrates, and the higher relative abundance of parasites potentially contributed to the high animal diversity (Ellis et al., 2015; Mahe et al., 2017). Furthermore, some parasites are important plant pathogens that can cause fatal diseases (Geisen et al., 2018). Replacing chemical-only fertilization with organic fertilization is widely considered a possible approach for maintaining healthy soil functioning ecosystems that can effectively inhibit plant diseases (Liu et al., 2013). Water is a dominant factor in protist dispersal. As shown by the PLS-PM results, SM had direct and significant effects on the parasite groups (Figure 5). The cysts and oocysts could withstand desiccation and survive in soil for very long periods of time, even many years (Shmakova et al., 2016).

Photosynthetic protists mainly contributed to SOC sequestration as the primary production (Jassey et al., 2015). We observed an increased abundance of photosynthetic protists under the MNPK treatment compared to the NPK treatment (Figure 2A). The PLS-PM indicated that SM directly affected the phototrophic structure of protists, which was supported by the results based on global research on protists (Oliverio et al., 2020). They explained the results based on the basic ecology of protists, given that most protistan lineages require water to move, feed and reproduce (Lentendu et al., 2014). Organic fertilizer application was considered an effective practice to maintain the soil water content (Korodjouma et al., 2006; Kiboi et al., 2019), which further explained the higher relative abundance observed under the MNPK treatment. Moreover, Jassey et al. (2015) reported that photosynthetic mixotrophic protists contributed significantly to carbon sequestration in carbon-rich peatland soils. However, the role of mixotrophic protists in carbon sequestration has not been characterized; hence, further studies are necessary to address this limitation.

Protists are an essential component of the soil food web, and further evaluating the main drivers of functional groups of protists is of major importance for ecosystem stability and ecosystem services (Geisen et al., 2017). Our study provided novel evidence that protistan functional groups respond differently to biotic and abiotic factors in the fertilization agroecosystem. The results imply that soil protist functional groups have specific biotic and abiotic regulation mechanisms under different fertilization management measures. Although preliminary, these findings advance our functional knowledge of soil protists and their driving factors, helping to further forecast the responses and functions of the protist community with different fertilization practices in agroecosystems. In soil food webs, top-down control (e.g., bacteriophages affected the bacterial community (Li et al., 2019) also occurred in community construction, and it is necessary to incorporate more realistic soil food web models in future research. In addition, it also should be noted that the primer limitation caused the missing of some important plant-associated groups (Fiore-Donno et al., 2018; Sapp et al., 2019), and advanced tools for metabarcoding are needed to further soil protistology research.

## Conclusion

Our study provides insights into the diversity and community structure of protists and expands our understanding of how fertilization regimes can shape soil protistan functional groups by regulating edaphic and abiotic (bacterial and fungal community composition) factors in a 5-year fertilization agricultural system. The results suggested that fertilization significantly changed the protistan community composition and the relative abundance of some taxa. At the supergroup level, Archaeplastida, Alveolata and Hacrobia were most sensitive to fertilization. The consumers, parasites and phototrophic composition of protists showed remarkable responses to fertilization and were derived from the distinct impacts of biotic and abiotic factors under the four fertilization regimes. Consumers, which were not sensitive to changes in abiotic factors, were mainly impacted by the fungal community composition. SM and AP were the main drivers shaping the phototrophs and parasites, respectively. These findings advance our knowledge on the impacts of driving factors in regulating functional groups of protists, facilitating sustainable agriculture through the manipulation of the protistan communities.

## Data availability statement

The original contributions presented in the study are included in the article/Supplementary material; further inquiries can be directed to the corresponding authors.

## Author contributions

SZ: writing—original draft, experiments, data curation, methodology, software, formal analysis, and visualization. HW, HaiZ, GL, HL, and GZ: grammar checking. WX: experiments and project administration. ZZ, HaoZ, and NJ: data curation. DY and JZ: experiments, resources, writing review and editing, and supervision. All authors contributed to the article and approved the submitted version.

## Conflict of interest

The authors declare that the research was conducted in the absence of any commercial or financial relationships that could be construed as a potential conflict of interest.

## Publisher’s note

All claims expressed in this article are solely those of the authors and do not necessarily represent those of their affiliated organizations, or those of the publisher, the editors and the reviewers. Any product that may be evaluated in this article, or claim that may be made by its manufacturer, is not guaranteed or endorsed by the publisher.

## Supplementary material

The Supplementary material for this article can be found online at: https://www.frontiersin.org/articles/10.3389/fmicb.2022.1036362/full#supplementary-material
