# Supplementary material for: Fertilization drives distinct biotic and abiotic factors in regulating functional groups of protists in a 5-year fertilization system
Source: Front Microbiol. 2022 Dec 5;13:1036362. doi: 10.3389/fmicb.2022.1036362 (PMC9760849; doi:10.3389/fmicb.2022.1036362)

**Supplemental materials**

**Table S1** Soil edaphic factors under different fertilization treatments. Means ± standard deviations (*n*=6). Different letters indicate statistical significance at the *P*<0.05 level based on Duncan’s test. SOC, soil organic carbon; TN, total nitrogen; TP, total phosphorus; AP, available phosphorus; DOC, dissolved organic carbon; NH_4_^+^-N, ammonia nitrogen; NO_3_^—^N, nitrate nitrogen. CK, no fertilization; M, organic fertilization; MNPK, combined organic and inorganic fertilization; NPK, inorganic fertilization.

| Treatment | pH | SOM  （g·kg^-1^） | SM  （%） | Total N  （g·kg^-1^） | Total P  （g·kg^-1^） | Available P  （mg·kg^-1^） | NH_4_^+^-N  （mg·kg^-1^） | NO_3_^—^N  （mg·kg^-1^） | DOC  (mg·kg^-1^) |
| --- | --- | --- | --- | --- | --- | --- | --- | --- | --- |
| CK | 8.42±0.02a | 20.55±0.26d | 21.76±0.61c | 1.28±0.02c | 0.73±0.01c | 12.87±0.48c | 1.46±0.05c | 6.57±0.42d | 125.66±2.33c |
| M | 7.76±0.06c | 28.41±0.31a | 26.44±0.48b | 1.58±0.02a | 1.09±0.02a | 42.94±3.64b | 1.60±0.04b | 9.23±0.45c | 177.08±4.67a |
| MNPK | 8.11±0.06b | 25.26±0.53b | 28.51±0.27a | 1.56±0.03a | 0.97±0.01b | 45.05±3.02b | 1.61±0.01b | 15.98±0.26b | 158.08±4.62b |
| NPK | 8.16±0.04b | 21.89±0.20c | 21.36±0.81c | 1.48±0.01b | 0.98±0.03b | 56.00±3.24a | 1.74±0.04a | 18.58±0.47a | 163.22±7.19ab |

**Table S2** Diversity of protist communities (Shannon, PD and observed indexes). Different letters indicate statistical significance at the *P*<0.05 level.

|  | CK | M | MNPK | NPK |
| --- | --- | --- | --- | --- |
| Shannon | 4.14±0.10a | 3.95±0.18a | 3.83±0.13a | 4.27±0.12a |
| PD | 68.97±3.55a | 71.91±1.01a | 69.80±5.31a | 72.90±2.51a |
| Observed | 386.33±30.24a | 372.17±17.61a | 350.00±31.98a | 342.17±11.02a |

**Table S3** Effects of fertilization on protistan β-diversity based on multivariate permutational analysis of variance (PERMANOVA). The PERMANOVA used distance matrixes. Values represent the pseudo-F (*F*) and the permutation-based level of significance (*P*).

|  | F | R^2^ | P | F | P |
| --- | --- | --- | --- | --- | --- |
| CK-M | 2.53 | 0.20 | 0.001 | 2.23 | <0.001 |
| CK-MNPK | 2.49 | 0.20 | 0.008 |  |  |
| CK-NPK | 2.87 | 0.22 | 0.003 |  |  |
| M-MNPK | 1.45 | 0.13 | 0.061 |  |  |
| M-NPK | 2.29 | 0.19 | 0.009 |  |  |
| MNPK-NPK | 1.79 | 0.15 | 0.031 |  |  |

**Table S4** Mantel tests used to estimate the effects of biotic and abiotic factors on the composition of the functional groups of protists.

|  | Functional community | | |
| --- | --- | --- | --- |
|  | Consumers | Phototrophs | Parasites |
| **Edaphic factors** |  |  |  |
| pH | 0.11 | 0.008 | 0.14 |
| SOM | **0.20*** | 0.08 | 0.02 |
| NO_3_^-^-N | 0.03 | 0.10 | **0.15*** |
| NH_4_^+^-N | 0.08 | 0.19 | **0.38**** |
| TN | 0.17 | 0.01 | 0.12 |
| TP | **0.32**** | 0.08 | 0.10 |
| AP | **0.20*** | 0.04 | **0.24*** |
| DOC | 0.04 | -0.04 | 0.15 |
| SM | 0.11 | **0.17*** | **0.19*** |
| **Biotic factors** |  |  |  |
| Bacterial community | **0.19*** | 0.12 | **0.22*** |
| Fungal community | **0.38**** | 0.02 | **0.27*** |

**Fig. S1** Relative abundance of the protist community at the supergroup level under different fertilization treatments. Error bars represent the standard deviations from the mean, *n*=6 for all values. Different letters on the bar of a panel indicate statistical significance at the *P*<0.05 level.


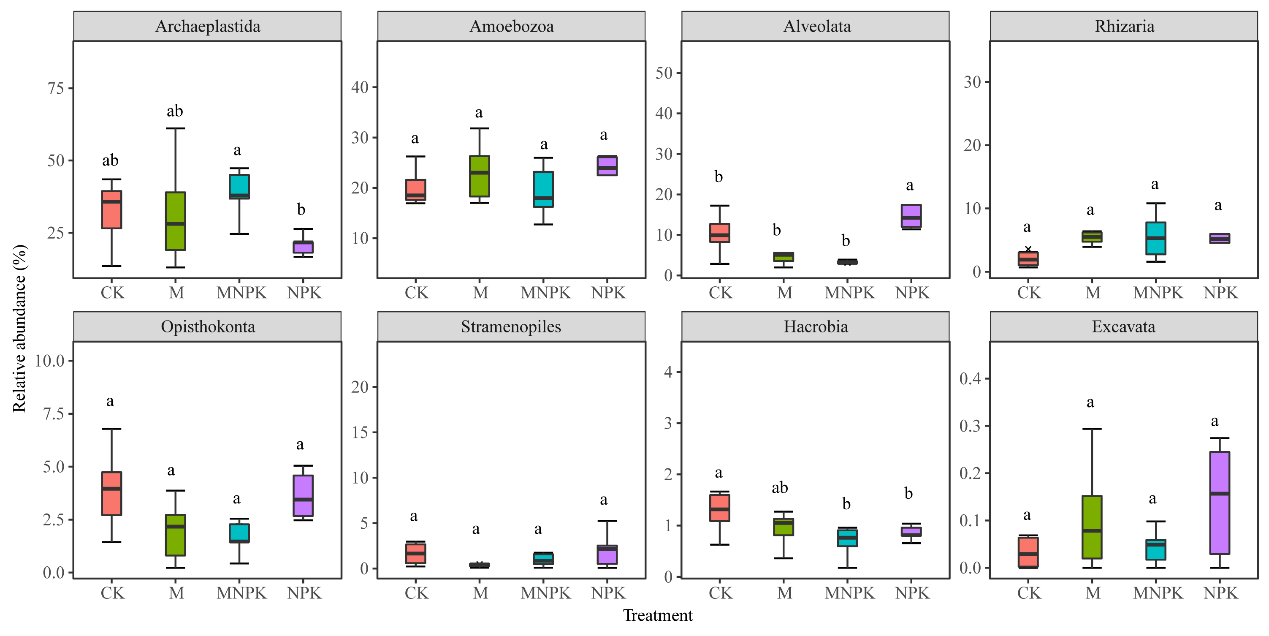

Supplement: Supplementary file 1 [file Data_Sheet_1.docx]
